# RNA-Seq Analyses for Two Silkworm Strains Reveals Insight into Their Susceptibility and Resistance to *Beauveria bassiana* Infection

**DOI:** 10.3390/ijms18020234

**Published:** 2017-02-10

**Authors:** Dongxu Xing, Qiong Yang, Liang Jiang, Qingrong Li, Yang Xiao, Mingqiang Ye, Qingyou Xia

**Affiliations:** 1 State Key Laboratory of Silkworm Genome Biology, Southwest University, Chongqing 400715, China; dongxuxing@126.com (D.X.); jiangliang@swu.edu.cn (L.J.); 2 Sericulture and Agri-Food Research Institute, Guangdong Academy of Agricultural Sciences, Guangzhou 510610, China; serilover@126.com (Q.Y.); liqr7702@aliyun.com (Q.L.); xiaoyang1209@163.com (Y.X.); yemingq@163.com (M.Y.)

**Keywords:** silkworm, RNA-seq, *Beauveria bassiana*, resistance

## Abstract

The silkworm *Bombyx mori* is an economically important species. White muscardine caused by *Beauveria bassiana* is the main fungal disease in sericulture, and understanding the silkworm responses to *B. bassiana* infection is of particular interest. Herein, we investigated the molecular mechanisms underlying these responses in two silkworm strains Haoyue (HY, sensitive to *B. bassiana*) and Kang 8 (K8, resistant to *B. bassiana*) using an RNA-seq approach. For each strain, three biological replicates for immersion treatment, two replicates for injection treatment and three untreated controls were collected to generate 16 libraries for sequencing. Differentially expressed genes (DEGs) between treated samples and untreated controls, and between the two silkworm strains, were identified. DEGs and the enriched Kyoto Encyclopedia of Genes and Genomes (KEGG) pathways of the two strains exhibited an obvious difference. Several genes encoding cuticle proteins, serine proteinase inhibitors (SPI) and antimicrobial peptides (AMP) and the drug metabolism pathway involved in toxin detoxification were considered to be related to the resistance of K8 to *B. bassiana.* These results revealed insight into the resistance and susceptibility of two silkworm strains against *B. bassiana* infection and provided a roadmap for silkworm molecular breeding to enhance its resistance to *B. bassiana*.

## 1. Introduction

The silkworm, *Bombyx mori*, is an important economic insect for silk production in many developing countries [1]. It is also a lepidopteran model insect in the investigation of insect genetics and immunology [2,3,4]. *Beauveria bassiana* is a major pathogenic fungus in sericulture. White muscardine disease caused by *B. bassiana* tends to break out in moist climate and leads to enormous damage [5].

In the natural environment, the process of *B. bassiana* infecting silkworms can be separated into three different stages: surface adhesion, cuticle penetration, and replication and host killing [6]. After cuticle penetration, the fungus is attacked by the innate immune responses of the silkworm [7], but the molecular mechanism of host response to *B. bassiana* infection remains poorly understood. Investigating the silkworm response to *B. bassiana* infection is of particular interest since it provides a roadmap for silkworm molecular breeding to enhance its resistance against *B. bassiana*. In addition, it gives useful information for genetic modification of the fungus to enhance its efficiency in pest control [8]. Hou et al. (2011) identified 77 differentially expressed genes (DEGs) involved in the mechanism of infection of *B. bassiana* using the suppression subtractive hybridization method [9]. In 2014, Hou et al. used digital gene expression profiling to probe the overall transcriptome of the Dazao silkworm strain during the early response against *B. bassiana* infection, and identified 1430 DEGs [10]. The results suggest many biological processes are involved in the interaction between the silkworm and *B. bassiana*. However, these studies only focused on one silkworm strain without considering differences between resistant and susceptible strains.

Haoyue (HY) is a bivoltine silkworm strain originating from Japan [11] and has the virtue of high silk yielding. By cross-breeding with Chinese strain Jingsong, the silkworm variety named Jingsong × Haoyue was cultivated and has been widely used in sericulture for many years. HY is highly sensitive to *B. bassiana* infection, in contrast to the Kang 8 (K8) strain that is highly resistant. K8 is a valuable germplasm resource preserved in Guangdong Sericultural Germplasm Bank and is well known for good healthiness. The molecular mechanisms underlying their different responses to *B. bassiana* infection remain unknown at present, and investigating differences in gene expression during *B. bassiana* infection may help us to understand the genes responsible for resistance in K8.

RNA-Seq is a revolutionary technology [12,13] providing an opportunity to study the transcriptional responses of K8 and HY to *B. bassiana* infection. In this study, the molecular mechanisms underlying the different responses to *B. bassiana* infection in HY and K8 were investigated via transcriptome analysis with the Illumina HiSeq 2500 platform. DEGs and their associated pathways were identified, and the results provide insight into the genes responsible for *B. bassiana* resistance and susceptibility.

## 2. Results

### 2.1. K8 and HY Survival Curve Analysis

**Figure 1:**
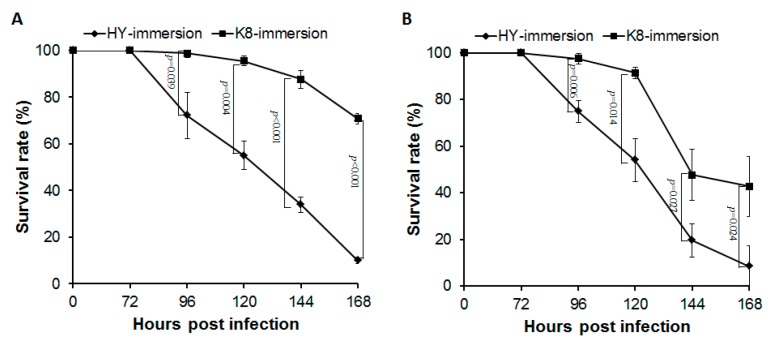
Survival curves of K8 and Haoyue (HY) strains infected by *B. bassiana*: (**A**) immersion treatment using a conidial suspension of 1 × 10^5^ conidia/mL; and (**B**) immersion treatment using a conidial suspension of 5 × 10^5^ conidia/mL. Error bars: SE of the mean. *p* represents the statistically significantly different value.

To verify the difference in resistance between the two silkworm strains, and choose the most critical time points for comparison, the survival rates of the two strains were monitored for 168 h after *B. bassiana* infection. As shown in Figure 1, the survival curves exhibited an obvious difference. When immersed in the conidial suspension (1 × 10^5^ conidia/mL), almost all HY silkworms were dead (survival rate ~9.93% ± 1.44%) at 168 hpi, whereas nearly 70% of the K8 silkworms were alive and able to spin cocoons (Figure 1A). Treatment with conidial suspension (5 × 10^5^ conidia/mL) showed similar result (Figure 1B). Generally, the survival time of K8 strain was much longer than HY strain. Most infected silkworms were able to spin cocoons, which effectively reduced the economic losses.

The sampling time for RNA-Seq was determined according to the survival curves. For HY, all the silkworms were alive at 72 hpi, but a significant decrease was apparent by 96 hpi, suggesting 72–96 hpi is the critical period for interaction with *B. bassiana* [14,15]. Meanwhile, K8 silkworms did not die in significant numbers until 96 hpi, and the overall survival rate was much higher, indicating clear differences in resistance strategies in response to *B. bassiana*. Thus, a time point of 96 hpi was chosen to investigate the transcriptional response of the silkworms.

Besides, the mortality rates of K8 and HY were also remarkably different following *B. bassiana* injection. The injection treatment was carried out at 86 h of fifth instar larvae. Thus, most of the silkworms did not die until spinning cocoons. The survival rates of K8 and HY were 75.56% and 42.22%, respectively, before cocooning, indicating resistance difference between the two strains.

### 2.2. Gene Expression in K8 and HY Strains

To compare differences between the transcriptomes of resistant and sensitive silkworm strains, cDNA libraries were generated from larvae of fifth instar, and then Illumina paired-end sequencing was performed. A total of ~12 M, 50-bp, single-ended RNA-Seq reads were generated from each sample. There were three biological replicates for control and immersion treatment respectively and two replicates for injection treatment each for K8 and HY. As shown in Table 1, the average clean read number was 14,586,874. Each of the reads was mapped to the silkworm genome sequence, which contains 18,510 predicted genes [16]. All of the samples showed similar match results, with ~89% of reads matching the predicted genes of the genome and ~57% being unique matches. Besides, pearson correlation among samples (Figure S1) and scatter plot of gene expression of biological repeats (Figure S2) all revealed that the samples selection is reasonable, with good correlations among biological replicates, and the sequencing data could be used for following analyses.

### 2.3. Analysis of DEGs in HY and K8 Strains

**Figure 2:**
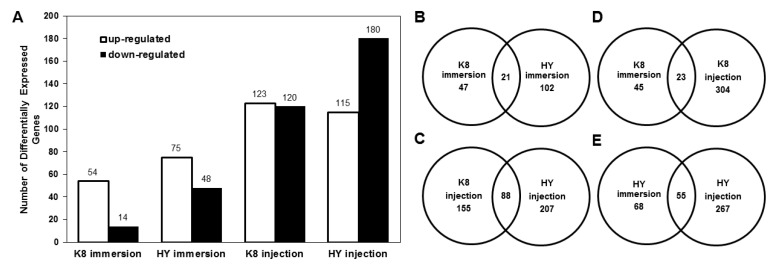
Statistics of differentially expressed genes (DEGs) in K8 and HY strains: (**A**) number of up-regulated and down-regulated DEGs; (**B**) Venn plot of DEGs in K8 and HY in the immersion experiment; (**C**) Venn plot of DEGs in K8 and HY in the injection experiment; (**D**) Venn plot of DEGs in K8 in immersion and injection experiments; and (**E**) Venn plot of DEGs in HY in immersion and injection experiments.

To explore differences in gene expression in HY and K8 silkworm strains in response to *B. bassiana*, immersion experiments were performed to identify DEGs between HY immersion samples and HY controls, and between K8 immersion samples and K8 controls. We then compared the differences between immersion response genes in HY and K8 strains. The same analyses were then performed for the injection experiment. As shown in Figure 2A and Table S3, in the immersion experiment, 68 DEGs were identified in K8, including 54 up-regulated genes and 14 down-regulated genes. For HY, 123 DEGs were detected, including 75 up-regulated genes and 48 down-regulated genes. The Venn plot revealed that 21 genes were differentially expressed in both K8 and HY (Figure 2B). In the injection experiment, 243 DEGs were detected in K8, including 123 up-regulated genes and 120 down-regulated genes. For HY, 295 DEGs were identified, including 115 up-regulated genes and 180 down-regulated genes. As shown in Figure 2C, 88 genes were differentially expressed in both K8 and HY.

We also compared DEGs identified in immersion and injection experiments. As shown in Figure 2D, 23 genes were consistently differentially expressed in K8 in both the immersion and injection experiments, whereas 55 genes were consistently differentially expressed in both the immersion and injection experiments in HY (Figure 2E).

**Figure 3:**
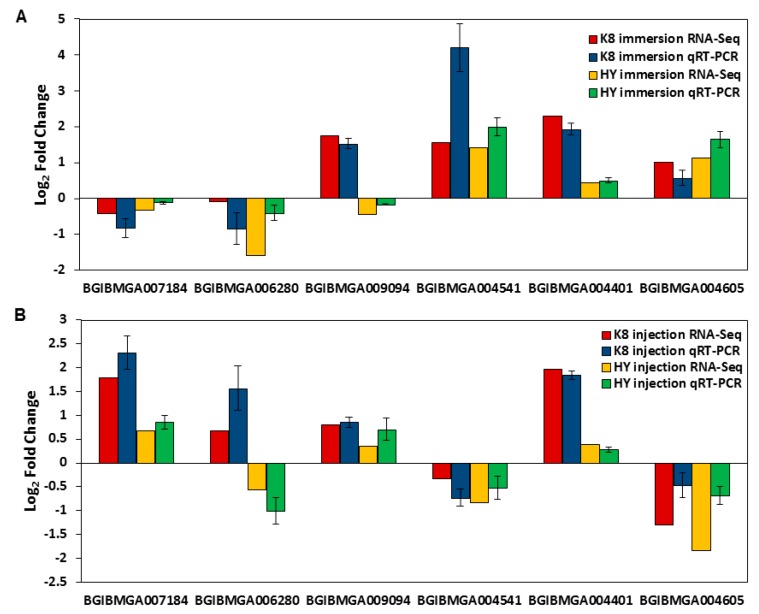
Verification of RNA-Seq results by qPCR: (**A**) expression patterns of six randomly selected DEGs identified in the immersion experiment; and (**B**) expression patterns of six randomly selected DEGs identified in the injection experiment. BGIBMGA007184: cytochrome P450 protein. BGIBMGA006280: cecropin A. BGIBMGA009094: BmSPI38. BGIBMGA004541: heat shock protein hsp20.4. BGIBMGA004401: 30K lipoprotein 19G1 precursor. BGIBMGA007184: heat shock protein hsp20.8.

To validate the gene expression data obtained using RNA-Seq, six DEGs were randomly selected for a quantitative real-time PCR (qPCR) analysis. The results (Figure 3) essentially confirmed that the qPCR data were consistent with the RNA-Seq data, indicating that the RNA-Seq approach was able to provide reliable differential gene expression data for this system.

### 2.4. Functional Classifications and Comparison of DEGs in K8 and HY Strains

**Figure 4:**
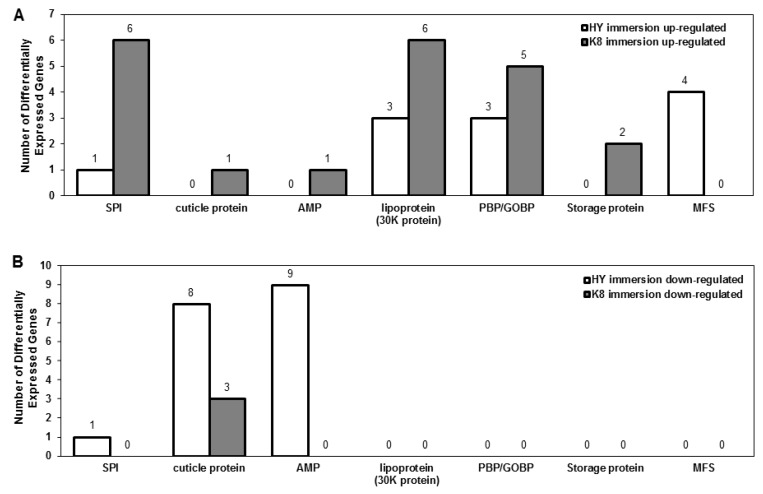
Functional classification of DEGs identified in the immersion experiment: (**A**) up-regulated genes; and (**B**) down-regulated genes. SPI, serine protease inhibitor; lipoprotein (30K protein), lepidopteran low molecular weight lipoprotein (30K protein); PBP/GOBP, insect pheromone/odorant binding protein; AMP, antimicrobial peptide; MFS, major facilitator superfamily; PGRP, peptidoglycan recognition protein; PBP/GOBP, pheromone binding protein/general odorant binding protein.

To better understand the identified DEGs, we classified them according to their biological functions (Table S1). In the immersion experiment, a large number of serine protease inhibitors (SPI), as well as lepidopteran low molecular weight lipoprotein (30K protein) and insect pheromone/odorant binding protein (PBP/GOBP) were up-regulated in K8. Genes encoding storage proteins, cuticle proteins, and antimicrobial peptides (AMP) were also specifically up-regulated in K8, while major facilitator superfamily (MFS) genes were specifically up-regulated in HY (Figure 4A). A greater number of genes encoding cuticle proteins, SPI and AMP were down-regulated in HY (Figure 4B).

**Figure 5:**
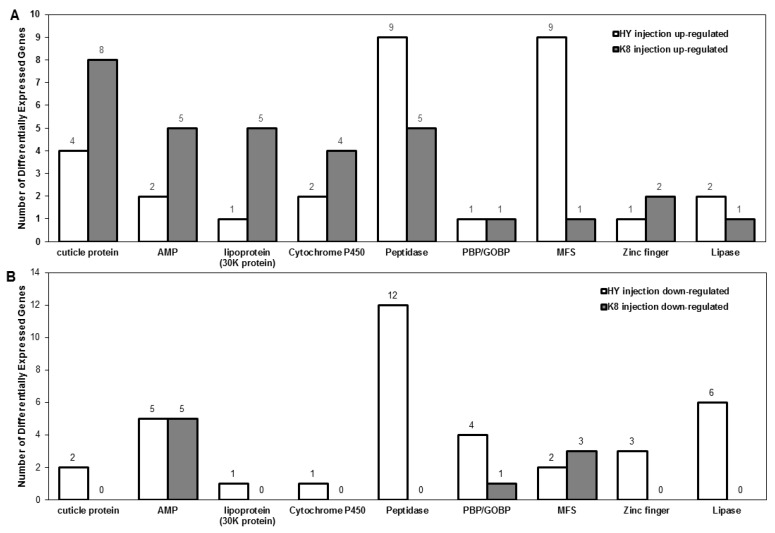
Functional classification of DEGs identified in the injection experiment: (**A**) up-regulated genes; and (**B**) down-regulated genes. Lipoprotein (30K protein), lepidopteran low molecular weight lipoprotein (30K protein); PBP/GOBP, insect pheromone/odorant binding protein; AMP, antimicrobial peptide; MFS, major facilitator superfamily.

In the injection experiment, a greater number of genes encoding cuticle protein, AMP, lipoprotein (30K protein), and cytochrome P450 were up-regulated in K8, while more genes encoding peptidases and MFS proteins were up-regulated in HY (Figure 5A). A larger number of genes with the functions of coding PBP/GOBP were down-regulated in HY, and genes encoding cuticle proteins, peptidases, lipoprotein (30K protein), cytochrome P450, zinc finger, and lipase were specifically down-regulated in HY (Figure 5B).

Pathway enrichment analysis was performed on DEGs identified in each group (Table 2 and Table S4). For the immersion experiment, five and eight pathways were significantly enriched with DEGs in K8 and HY, respectively (*p* < 0.05), and no common pathway was detected in these two strains. For the injection experiment, 40 and 32 pathways were significantly enriched with DEGs in K8 and HY, respectively (*p* < 0.05), and 19 pathways were enriched with DEGs in both strains. In K8, the pancreatic secretion pathway, which was related to digestion and abortion of nutrition, was clearly enriched in both immersion and injection experiments. In HY, “phagosome”, “lysosome”, “glycerolipid metabolism” and “ECM-receptor interaction” pathways were enriched in both immersion and injection experiments.

### 2.5. Comparison of DEGs between HY and K8 Strains

**Figure 6:**
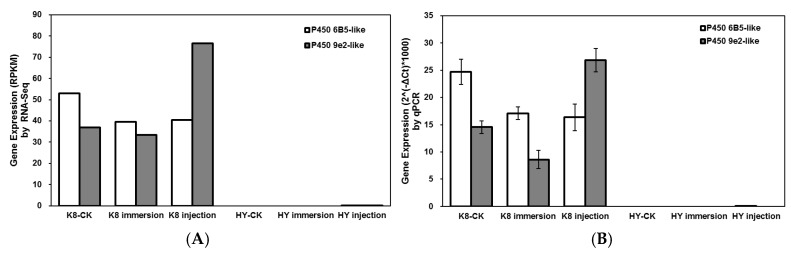
Validation of K8-specific expressed genes by qPCR: (**A**) RNA-Seq results of *P450 6B5-like* and *P450 9e2-like*; and (**B**) qPCR results of *P450 6B5-like* and *P450 9e2-like*.

The sensitivity to *B. bassiana* is distinct in the two silkworm strains, and the genes that are differentially expressed between HY and K8 strains may be responsible for the dissimilar responses to the fungal pathogen. We therefore compared DEGs between HY and K8 strains. HY and K8 samples consisted of three control samples, three immersion samples and two injection samples, respectively. A total of 260 DEGs were detected, including 116 up-regulated genes and 144 down-regulated genes in K8 (*q* < 0.05 and log_2_ (Fold change) ≥ 1, deSeq). As shown in Table 3, three pathways, including the RIG-I-like receptor signaling pathway, drug metabolism—other enzymes and retinol metabolism pathway were enriched with these DEGs. Among these DEGs, we further selected K8-specific genes with the following standards: RPKM ≥ 1 in each K8 sample; mean RPKM in HY < 0.1; log_2_ (Fold change) ≥ 3. Under the standards, six genes could be considered as K8-specific genes (Table 4), including three genes in the cytochrome P450 family, a heat shock protein, bmp-2, and a quinoprotein amine dehydrogenase. The qPCR analysis confirmed that the genes *P450 6B5-like* and *P450 9e2-like* were specifically expressed in K8 strain (Figure 6).

## 3. Discussion

In early stage, we identified two silkworm strains, K8 and HY; K8 was highly resistant to *B. bassiana* while HY was very sensitive. In this study, we carried out RNA-Seq on silkworm K8 and HY strains to investigate the reasons for their distinct susceptibility to *B. bassiana* using immersion and injection treatments. In the immersion experiment, 68 and 123 DEGs were identified in K8 and HY respectively, of which 21 genes were commonly differentially expressed. KEGG pathway enrichment analysis indicated that five and eight pathways are significantly enriched in K8 and HY respectively, and no common pathway is detected. In the injection experiment, 243 and 295 DEGs were identified in K8 and HY respectively and only 88 genes were common. Besides, 40 and 32 pathways were significantly enriched in K8 and HY respectively and only 19 pathways were common. The results above showed that the responses against *B. bassiana* infection were different in the two silkworm strains.

We classified DEGs according to their biological functions and compared the difference of response genes between HY and K8. The results showed that cuticle protein-encoding genes were significantly differentially expressed in K8 and HY strains. The cuticle serves as an effective physical barrier against potential pathogenic microorganisms and many toxic substances. After exposure to pesticides, the resistant strains of insects can increase the gene expression of cuticle proteins that stop pesticides from penetrating [17,18,19]. In the immersion experiment, the gene encoding cuticle protein was significantly up-regulated in K8, while a number of genes encoding cuticle proteins in the HY strain were down-regulated. This indicates that the synthesis of cuticle proteins may be inhibited in HY upon penetration by *B. bassiana*. Cuticle proteins have also been linked to wound healing in insects [20], possibly explaining the up-regulation of cuticle proteins in both K8 and HY strains in the injection experiment. Even accounting for this, the number of up-regulated genes was still twice as high in the K8 strain. We therefore concluded that K8 silkworms might synthesize more cuticle proteins that stop *B. bassiana* penetrating the hemocoel.

Studies have shown that serine protease inhibitors play important roles in insect immunity [21,22,23]. During the invasion process, the fungi can secrete abundant extracellular proteases to penetrate the host physical barriers [24]. To resist invasion by pathogens, some hosts produce a large amount of protease inhibitors in response to microbial proteases [25,26]. Zhao et al. found that at least 19 SPI genes were up- or down-regulated in silkworm following infection [27], indicating a role in resistance to pathogenic microorganisms. In the immersion experiment, a larger number of SPI genes such as BmSPI38 (BGIBMGA009094), protease inhibitor 4 (BGIBMGA008711) and kazal-type proteinase inhibitor (BGIBMGA011573) were up-regulated in K8 strain. BmSPI38 and BmSPI39 could inhibit the germination of *B. bassiana* conidia and markedly improve the survival rate of silkworm hosts [28,29]. The up-regulated SPIs in the K8 strain may help to inhibit the germination of *B. bassiana* conidia and thus delay or prevent invasion.

AMPs and 30K proteins were also related to insect immunity [10]. Due to lack of an adaptive immune system in insects, AMPs play a crucial role in the struggle against pathogens [30]. The genes *cecropin A*, *cecropin B* and *enbocin1* have been shown to have high antifungal activity [31,32,33]. In the immersion experiment, six AMP genes such as cecropin A, cecropin D, gloverin-like protein 4, moricin I were specifically down-regulated in HY. The 30K proteins also play a role in protection of *B. mori* against invading fungi [34]. Compared to HY, a greater number of 30K proteins were up-regulated in K8. The up-regulated 30K lipoproteins 19G1 precursor may participate in the pathogen recognization of the silkworms by bounding to glucose and glucans [35]. However, as a major class of proteins involved in drug resistance [36], MFS genes were significantly up-regulated in HY. MFS transporters could pump the drugs into the extracellular and have been found to be the main mechanism of multidrug-resistance in bacteria and fungi [37,38]. Whether MFS transporters acted in the detoxification of *B. bassiana* toxin remains unstudied.

Furthermore, DEGs of eight HY samples and eight K8 samples were also compared. Six genes were specifically expressed in K8, including three encoding cytochrome P450 family proteins. Two of these (*P450 6B5-like and P450 9e2-like*) were selected for interrogation by qPCR, the results of which confirmed their specific expression in the K8 strain. Cytochrome P450 proteins function as a major class of detoxification enzymes [39], and most insecticide-resistant genes belong to the CYP4, CYP6 and CYP9 subfamilies [40,41,42]. Interestingly, two of the genes specifically expressed in K8 belong to the CYP6 and CYP9 classes, suggesting they may play roles in resistance to *B. bassiana* infection.

KEGG pathway analyses identified three pathways enriched with DEGs between K8 and HY, including drug metabolism-other enzymes, RIG-I-like receptor signaling pathway, and retinol metabolism. The genes of the drug metabolism pathway, including aldehyde oxidase (BGIBMGA008269), carboxylesterase (BGIBMGA012122), and UDP-glucuronosyltransferase (BGIBMGA010294, BGIBMGA013830), were all up-regulated in the K8 strain. Similar to cytochrome P450, these enzymes are essential components of insect toxin detoxification pathway [43]. We propose that the cytochrome P450, carboxylesterase, and UDP-glucuronosyltransferase function effectively in the detoxification of toxins such as cyclic peptide toxins secreted by *B. bassiana*, which delayed the death of the K8 strain. Besides, genes in the RIG-I-like receptor signaling pathway can initiate immune activation in a variety of cell types [44] and this pathway is related to host immune response [45,46]. A previous study showed that this pathway is enriched with positive selected genes in the silkworm, which might be a result of the selection against pathogens during silkworm domestication [47].

The insect-fungus interactions have been studied for several years. However, the mechanisms of resistance remain poorly understood, and many of the key genes and proteins involved have not been established. In the present study, we investigated host response to *B. bassiana* infection in highly susceptible (HY) and highly resistant (K8) strains using an RNA-seq approach. The results revealed that a larger number of genes encoding cuticle protein, SPI, AMP and 30K protein were up-regulated in the K8 strain and down-regulated in the HY strain. Moreover, a series of detoxification-related genes were specifically up-regulated in the K8 strain. It could be speculated further that K8 silkworms could increase level of cuticle protein and SPI, which thereby inhibited the germination and penetration of *B. bassiana* conidia, and then activate the drug metabolism pathway to remove the toxins secreted by *B. bassiana*, ultimately resulted in high resistance to *B. bassiana*. Further research on the exact mechanisms of these genes is needed.

## 4. Materials and Methods

### 4.1. Silkworm Strains and Fungal Strain

The trial was approved by the Experimental Animal Ethics Committee of Sericulture and Agri-Food Research Institute, Guangdong Academy of Agricultural Sciences (25 March 2015, approval number: B2015-02). The silkworm strains HY (sensitive to *B. bassiana*) and K8 (resistant to *B. bassiana*) were provided by the Guangdong Sericultural Germplasm Bank (Guangzhou, China). Larvae were reared on fresh mulberry leaves at 27 °C, and newly exuviated larvae of the fifth instar were used in all experiments.

The fungal strain *B. bassiana* (Genbank ID: KM205065), isolated from silkworm larvae, was preserved at 4 °C on slants of potato dextrose agar. Conidia, harvested from cultures grown for 7 days at 26 °C and 95% relative humidity, were suspended in sterile distilled water for experiments.

### 4.2. Survival Curves Assay of K8 and HY Strains

For each silkworm strain, newly exuviated larvae of fifth instar were immersed in the conidial suspension (1 × 10^5^ and 5 × 10^5^ conidia/mL) for 15 s, respectively [10]. Immersed silkworms were reared at 26 °C and 95% relative humidity, and the number of living silkworms was recorded daily until they started spinning cocoons. The dead silkworms were moved to another plastic box for incubation to confirm the infection by *B. bassiana*. For the control group, the silkworms were immersed in sterile distilled water for the same period. Each assay was repeated four times.

### 4.3. Preparation of Silkworm Samples for Sequencing

Newly exuviated larvae of the fifth instar were immersed in the conidial suspension (5 × 10^5^ conidia/mL) as described above. At 96 h post infection (hpi), three independent larvae of each strain were collected respectively. For eliminating the effect of the body wall on *B. bassiana* defenses in K8 and HY strains, injection experiments were also performed. Since 10 h post injection has been shown as an appropriate time point for investigating the insect responses to *B. bassiana* conidia in previous reports [9,48], 2 µL of diluted conidial suspension (1 × 10^7^ conidia/mL) were injected into the hemocoel of *Bombyx mori* fifth instar 86 h larvae, and 10 h later, two independent larvae of each strain were collected for subsequent sequencing experiments. Untreated fifth-instar larvae were used as the control group at 96 h. All silkworm samples were therefore collected at the same developmental stage (96 h post exuviation) to eliminate the influence of development on gene expression [14,15].

An independent larva was used as a sample. A total of 16 samples were subjected to sequencing, including three K8 control samples, three K8 immersion samples, two K8 injection samples, three HY control samples, three HY immersion samples and two HY injection samples.

### 4.4. RNA Extraction, Illumina Sequencing, and Data Processing

Total RNA from each sample was extracted using Trizol reagent (Life Technologies, Carlsbad, CA, USA) according to the manufacturer’s protocol. RNA purity was assessed by using an ND-1000 NanoDrop (Thermo Scientific, Wilmington, DE, USA). Each RNA sample had an A260:A280 ratio above 1.8 and an A260:A230 ratio above 2.0. RNA integrity was evaluated using the Agilent 2200 Tape Station (Agilent Technologies, Santa Clara, CA, USA), and all samples had an RNA integrity value above 7.0. Briefly, mRNAs were isolated from total RNA, fragmented to approximately 200 bp, and subjected to first strand and second strand cDNA synthesis followed by adaptor ligation and low-cycle enrichment according to the instructions of the TruSeq RNA LT/HT Sample Prep Kit (Illumina, San Diego, CA, USA).

Purified library products were evaluated using the Agilent 2200 Tape Station and Qubit 2.0 software (Life Technologies). RNA-Seq was performed at RiboBio Co., Ltd. (Guangzhou, China) using the HiSeq 2500 platform (Illumina, San Diego, CA, USA). Prior to sequencing, raw data were filtered to produce high-quality clean data, and all subsequent analyses were performed using clean data.

### 4.5. Annotation and Analysis of Sequence Data

For annotation, clean data were mapped to the silkworm transcriptome reference database using Soap2 [49]. Only reads with uniquely mapping to reference sequences were retained for subsequent analyses. Gene expression values were quantified as reads per kb of transcript per million mapped reads (RPKM) [50]. If two or more transcripts were detected for a gene, the longest transcript was retained to calculate its expression level. For each comparison group, DEGs were detected by DEGseq [51] with a false discovery rate <0.05 and an absolute log_2_ ratio ≥1. Pathways significantly enriched with DEGs were identified based on the Kyoto Encyclopedia of Genes and Genomes (KEGG) database and hypergeometric test (*q* < 0.05).

### 4.6. qPCR Analysis

To validate the results of DEG analysis, qPCR experiments were performed on a Roche LightCycler 480 system using SYBR-GREEN1 fluorescent reagents (TaKaRa, Otsu, Shiga, Japan). All designed primers were synthesized at Beijing Liuhe Huada Genomics Technology Co., Ltd. (Beijing, China) (Table S2). The *BmactinA3* gene was used as the endogenous control [52]. qPCR reactions (25 μL) consisted of a denaturation step at 95 °C for 10 min, followed by 40 cycles of 95 °C for 15 s, 55 °C for 15 s, and 72 °C for 45 s. Three biological replicates were performed for each gene, and relative fold changes were determined using the 2^−ΔΔ*C*t^ method [53].

## 5. Conclusions

In this study, we carried out RNA-Seq on silkworm K8 (resistant to *B. bassiana*) and HY (sensitive to *B. bassiana*) strains to investigate the reasons for their distinct susceptibility to *B. bassiana*. Different responses against *B. bassiana* infection were observed. Several genes encoding cuticle proteins, SPI and AMP and the drug metabolism pathway involved in toxin detoxification were considered to be related to the resistance of K8 to *B. bassiana*. Further molecular mechanism-based studies of host resistance to fungus are still needed.

## Figures and Tables

**Table 1:** Summary of sequencing data.

Sample	Raw Reads	Clean Reads	Clean Base	Total Mapped	Total Mapped Ratio	Unique Match
HY-control-1	14,538,847	14,283,926	714,196,300	12,821,136	89.76%	48.20%
HY-control-2	13,093,162	12,957,649	647,882,450	11,439,009	88.28%	53.63%
HY-control-3	14,750,894	14,604,137	730,206,850	12,986,072	88.92%	62.05%
HY-immersion-1	18,735,786	18,318,940	915,947,000	16,445,941	89.78%	52.31%
HY-immersion-2	14,899,363	14,591,964	729,598,200	13,070,880	89.58%	51.16%
HY-immersion-3	12,500,104	12,366,585	618,329,250	11,040,402	89.28%	54.10%
HY-injection-1	15,171,911	14,914,968	745,748,400	13,195,864	88.47%	74.54%
HY-injection-2	15,078,479	14,804,938	740,246,900	12,837,562	86.71%	72.23%
K8-control-1	17,655,181	17,298,316	864,915,800	15,429,685	89.20%	45.02%
K8-control-2	12,458,912	12,334,197	616,709,850	10,425,374	84.52%	57.16%
K8-control-3	13,424,038	13,277,104	663,855,200	11,612,998	87.47%	53.37%
K8-immersion-1	15,981,466	15,699,938	784,996,900	14,060,649	89.56%	40.49%
K8-immersion-2	17,412,127	17,068,312	853,415,600	15,368,966	90.04%	50.74%
K8-immersion-3	12,750,866	12,610,756	630,537,800	11,102,951	88.04%	47.15%
K8-injection-1	14,183,649	13,929,356	696,467,800	12,446,506	89.35%	74.64%
K8-injection-2	14,573,110	14,328,895	716,444,750	12,705,750	88.67%	74.15%
Average	14,825,493	14,586,874	729,343,691	12,936,859	88.60%	56.93%

**Table 2:** KEGG pathway enrichment analysis.

Pathway ID	Description	*p* Value			
		Immersion		Injection	
		K8	HY	K8	HY
ko00232	Caffeine metabolism	0.0045			
ko04974	Protein digestion and absorption	0.0266			
ko00020	Tricarboxylic acid (TCA) cycle	0.0304			0.0340
ko04972	Pancreatic secretion	0.0353		0.0410	
ko00720	Carbon fixation pathways in prokaryotes	0.0494			
ko00052	Galactose metabolism		0.0011		
ko04512	ECM-receptor interaction		0.0158		0.0214
ko00561	Glycerolipid metabolism		0.0221		0.0087
ko04145	Phagosome		0.0404	0.0006	3.9 × 10^−5^
ko04142	Lysosome		0.0452		0.0001
ko00603	Glycosphingolipid biosynthesis—globo series		0.0469		
ko00531	Glycosaminoglycan degradation		0.0469		
ko00604	Glycosphingolipid biosynthesis—ganglio series		0.0469		
ko04711	Circadian rhythm—fly			0.0004	0.0294
ko05130	Pathogenic Escherichia coli infection			0.0007	0.0119
ko00511	Other glycan degradation			0.0024	0.0023
ko05215	Prostate cancer			0.0041	0.0381
ko04919	Thyroid hormone signaling pathway			0.0062	0.0496
ko04390	Hippo signaling pathway			0.0088	
ko04975	Fat digestion and absorption			0.0105	
ko05166	Human T-cell lymphotropic virus I (HTLV-I) infection			0.0105	0.0486
ko04612	Antigen processing and presentation			0.0115	0.0109
ko05200	Pathways in cancer			0.0118	
ko05217	Basal cell carcinoma			0.0126	
ko00010	Glycolysis/Gluconeogenesis			0.0130	
ko05164	Influenza A			0.0138	0.0128
ko04151	PI3K-Akt signaling pathway			0.0155	0.0141
ko04391	Hippo signaling pathway—fly			0.0179	
ko04621	Nucleotide-binding oligomerization domain (NOD)-like receptor signaling pathway			0.0179	
ko00591	Linoleic acid metabolism			0.0186	
ko00565	Ether lipid metabolism			0.0192	
ko04141	Protein processing in endoplasmic reticulum			0.0199	0.0025
ko04510	Focal adhesion			0.0206	
ko04662	B cell receptor signaling pathway			0.0226	0.0214
ko03320	Peroxisome proliferator-activated receptor (PPAR) signaling pathway			0.0255	0.0242
ko05131	Shigellosis			0.0270	
ko00592	α-Linolenic acid metabolism			0.0270	
ko00600	Sphingolipid metabolism			0.0278	
ko05169	Epstein-Barr virus infection			0.0280	0.0260
ko05205	Proteoglycans in cancer			0.0284	
ko05222	Small cell lung cancer			0.0301	0.0285
ko04014	Ras signaling pathway			0.0306	
ko04540	Gap junction			0.0309	0.0293
ko05134	Legionellosis			0.0309	
ko04146	Peroxisome			0.0325	
ko04916	Melanogenesis			0.0333	
ko04144	Endocytosis			0.0352	
ko01040	Biosynthesis of unsaturated fatty acids			0.0419	
ko05160	Hepatitis C			0.0456	0.0433
ko04660	T cell receptor signaling pathway			0.0465	0.0441
ko04910	Insulin signaling pathway			0.0484	0.0459
ko04931	Insulin resistance				0.0055
ko05145	Toxoplasmosis				0.0062
ko05162	Measles				0.0094
ko00310	Lysine degradation				0.0118
ko04623	Cytosolic DNA-sensing pathway				0.0170
ko00380	Tryptophan metabolism				0.0181
ko05110	Vibrio cholerae infection				0.0201
ko04920	Adipocytokine signaling pathway				0.0221
ko05323	Rheumatoid arthritis				0.0285

**Table 3:** KEGG pathways containing genes differentially expressed in K8 and HY strains.

KEGG Term	*p*-Value	Corrected *p*-Value
ko04622:RIG-I-like receptor signaling pathway	0.0005	0.0459
ko00983:Drug metabolism—other enzymes	0.0012	0.0459
ko00830:Retinol metabolism	0.0012	0.0459

**Table 4:** Genes specifically expressed in the K8 strain.

Name	Length	KO Name	KO ID	Description
BGIBMGA004606	507	-	-	Heat shock protein hsp20.4 [Bombyx mori]
BGIBMGA004882	759	-	-	bmp-2 [Bombyx mori]
BGIBMGA013238	1347	-	-	P450 6B5-like|cytochrome P450 CYP6AE7 [Bombyx mori]
BGIBMGA003907	402	CYP3A	K07424	Cytochrome P450
BGIBMGA003908	1044	CYP9	K15003	P450 9e2-like|E-class P450, group II; Cytochrome P450
BGIBMGA010047	810	-	-	Quinoprotein amine dehydrogenase, β chain-like

## Supplementary Materials

Supplementary materials can be found at www.mdpi.com/1422-0067/18/2/234/s1.
