# Supplementary material for: RNA-Seq Analyses for Two Silkworm Strains Reveals Insight into Their Susceptibility and Resistance to Beauveria bassiana Infection
Source: Int J Mol Sci. 2017 Feb 10;18(2):234. doi: 10.3390/ijms18020234 (PMC5343773; doi:10.3390/ijms18020234)
Supplement: Supplementary file 1 [file ijms-18-00234-s001.zip › ijms-167379 suppl.pdf]

Supplementary Materials: RNA-Seq Analyses for Two Silkworm Strains Reveals Insight into Their Susceptibility and Resistance to *Beauveria bassiana* Infection

Dongxu Xing, Qiong Yang, Liang Jiang, Qingrong Li, Yang Xiao, Mingqiang Ye and Qingyou Xia

Table S1. Classification of the differently expressed genes (DEGs).

DEG Class	Immersion		Injection		Description
	HY	K8	HY	K8	
Peptidase	6	4	21	5	Peptidase
Lipoprotein	6	7	10	4	Lepidopteran low molecular weight lipoprotein
Protease inhibitor (PI)	2	6	8	8	Protease inhibitor
Major facilitator superfamily (MFS)	4	0	11	4	Major facilitator superfamily
cuticle protein	8	4	6	8	cuticle protein
Antimicrobial peptide (AMP)AMP	9	1	7	10	antimicrobial peptide
Pheromone binding protein/general odorant binding protein (PBP/GOBP)	3	5	5	2	Insect pheromone/odorant binding protein
Kinase	3	1	4	6	kinase
Glycoside hydrolase	4	1	4	2	Glycoside hydrolase
Tubulin	3	0	6	8	tubulin
Lipase	3	1	8	1	Lipase
Short-chain dehydrogenase/reductase (SDR)	0	0	6	2	Short-chain dehydrogenase/reductase
Cytochrome P450	0	0	3	4	Cytochrome P450
Immunoglobulin (Ig)	1	0	5	3	Immunoglobulin
Heat shock protein (HSP)	3	2	2	3	Heat shock protein
Zinc finger	0	0	4	2	Zinc finger
Cytochrome	1	0	1	3	Cytochrome
Cellular retinaldehyde-binding protein	0	0	4	1	Cellular retinaldehyde-binding protein
Carboxylesterase	0	0	3	3	Carboxylesterase
Glycosyl hydrolase	2	0	2	1	Glycosyl hydrolase
Epidermal growth factor (EGF)	0	0	3	3	Epidermal growth factor
Chorion protein	1	1	4	3	chorion protein
Chitin binding Peritrophin-A	2	1	0	2	Chitin binding Peritrophin-A
ATPase	1	0	2	2	ATPase
Peptidoglycan recognition protein (PGRP)	1	1	1	1	Peptidoglycan recognition protein
Juvenile hormone	2	0	1	0	juvenile hormone
Storage protein	0	2	1	1	Storage protein
Haem peroxidase	0	2	0	3	Haem peroxidase
Cyclin	1	1	3	2	Cyclin
Transketolase	2	0	0	0	Transketolase
Sterile	0	0	2	0	Sterile
Ribosomal protein	0	0	1	1	Ribosomal protein
Fibronectin	1	0	1	0	Fibronectin
Fibroin	0	0	0	2	fibroin
Ecdysone oxidase	0	1	0	2	Ecdysone oxidase
Dynein light chain LC8-type (DYNLL)	0	0	1	1	dynein light chain LC8-type
Cytosolic fatty-acid binding	0	0	2	0	Cytosolic fatty-acid binding
C-type lectin	0	0	1	1	C-type lectin
Caspase	0	0	1	1	caspase
Carbonic anhydrase	0	0	2	1	Carbonic anhydrase
Aminotransferase	0	0	1	1	Aminotransferase
Aldehyde dehydrogenase	0	0	1	1	Aldehyde dehydrogenase
Acyltransferase	0	0	2	1	Acyltransferase

Table S2. Quantitative reverse transcription PCR (qRT-PCR) primers and results.

Table S3. List of differentially expressed genes.

Table S4. Results of Kyoto Encyclopedia of Genes and Genomes (KEGG) pathway enrichment analysis.

	HY-CK-1	HY-CK-2	HY-CK-3	HY-imm-1	HY-imm-2	HY-imm-3	HY-stab-1	HY-stab-2	K8-CK-1	K8-CK-2	K8-CK-3	K8-imm-1	K8-imm-2	K8-imm-3	K8-stab-1	K8-stab-2
HY-CK-1	1	0.92	0.91	0.95	0.89	0.91	0.89	0.88	0.87	0.74	0.84	0.86	0.85	0.81	0.82	0.85
HY-CK-2	0.92	1	0.98	0.9	0.85	0.97	0.93	0.92	0.89	0.83	0.87	0.89	0.89	0.87	0.86	0.84
HY-CK-3	0.91	0.98	1	0.85	0.81	0.95	0.93	0.94	0.88	0.86	0.89	0.86	0.87	0.88	0.85	0.86
HY-imm-1	0.95	0.9	0.85	1	0.94	0.92	0.86	0.81	0.89	0.67	0.81	0.9	0.89	0.78	0.84	0.83
HY-imm-2	0.89	0.85	0.81	0.94	1	0.88	0.88	0.77	0.92	0.65	0.81	0.93	0.94	0.79	0.86	0.84
HY-imm-3	0.91	0.97	0.95	0.92	0.88	1	0.94	0.93	0.93	0.85	0.92	0.93	0.89	0.91	0.9	0.87
HY-stab-1	0.89	0.93	0.93	0.86	0.88	0.94	1	0.94	0.92	0.81	0.89	0.92	0.89	0.89	0.92	0.89
HY-stab-2	0.88	0.92	0.94	0.81	0.77	0.93	0.94	1	0.86	0.93	0.93	0.83	0.8	0.93	0.84	0.87
K8-CK-1	0.87	0.89	0.88	0.89	0.92	0.93	0.92	0.86	1	0.78	0.9	0.99	0.93	0.89	0.95	0.89
K8-CK-2	0.74	0.83	0.86	0.67	0.65	0.85	0.81	0.93	0.78	1	0.95	0.75	0.71	0.95	0.74	0.81
K8-CK-3	0.84	0.87	0.89	0.81	0.81	0.92	0.89	0.93	0.9	0.95	1	0.88	0.85	0.99	0.89	0.94
K8-imm-1	0.86	0.89	0.86	0.9	0.93	0.93	0.92	0.83	0.99	0.75	0.88	1	0.95	0.88	0.94	0.87
K8-imm-2	0.85	0.89	0.87	0.89	0.94	0.89	0.89	0.8	0.93	0.71	0.85	0.95	1	0.84	0.91	0.88
K8-imm-3	0.81	0.87	0.88	0.78	0.79	0.91	0.89	0.93	0.89	0.95	0.99	0.88	0.84	1	0.89	0.92
K8-stab-1	0.82	0.86	0.85	0.84	0.86	0.9	0.92	0.84	0.95	0.74	0.89	0.94	0.91	0.89	1	0.94
K8-stab-2	0.85	0.84	0.86	0.83	0.84	0.87	0.89	0.87	0.89	0.81	0.94	0.87	0.88	0.92	0.94	1

Figure S1. Pearson correlation among samples. Different colors indicate the degree of correlation among samples.

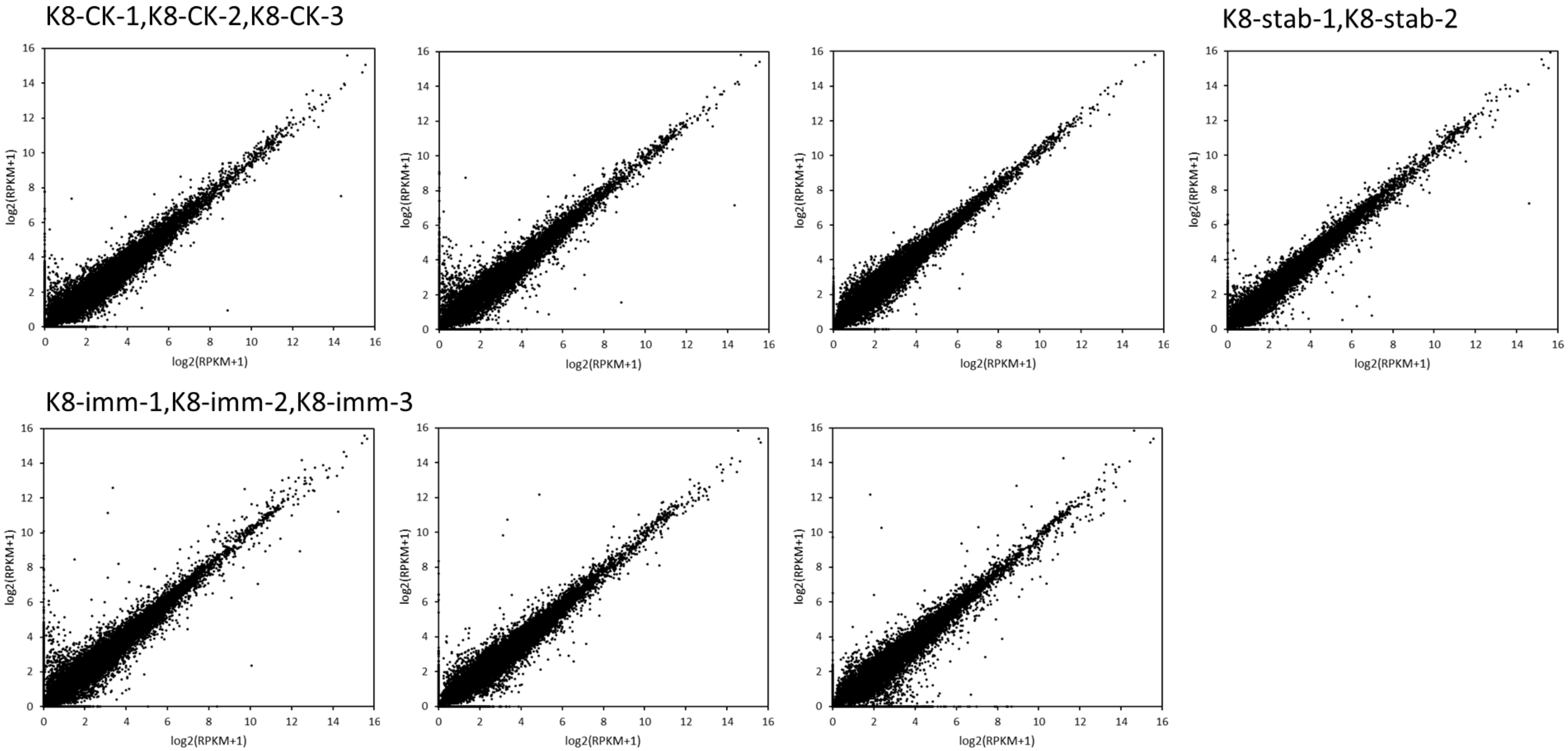

Figure 2. *Cont.*

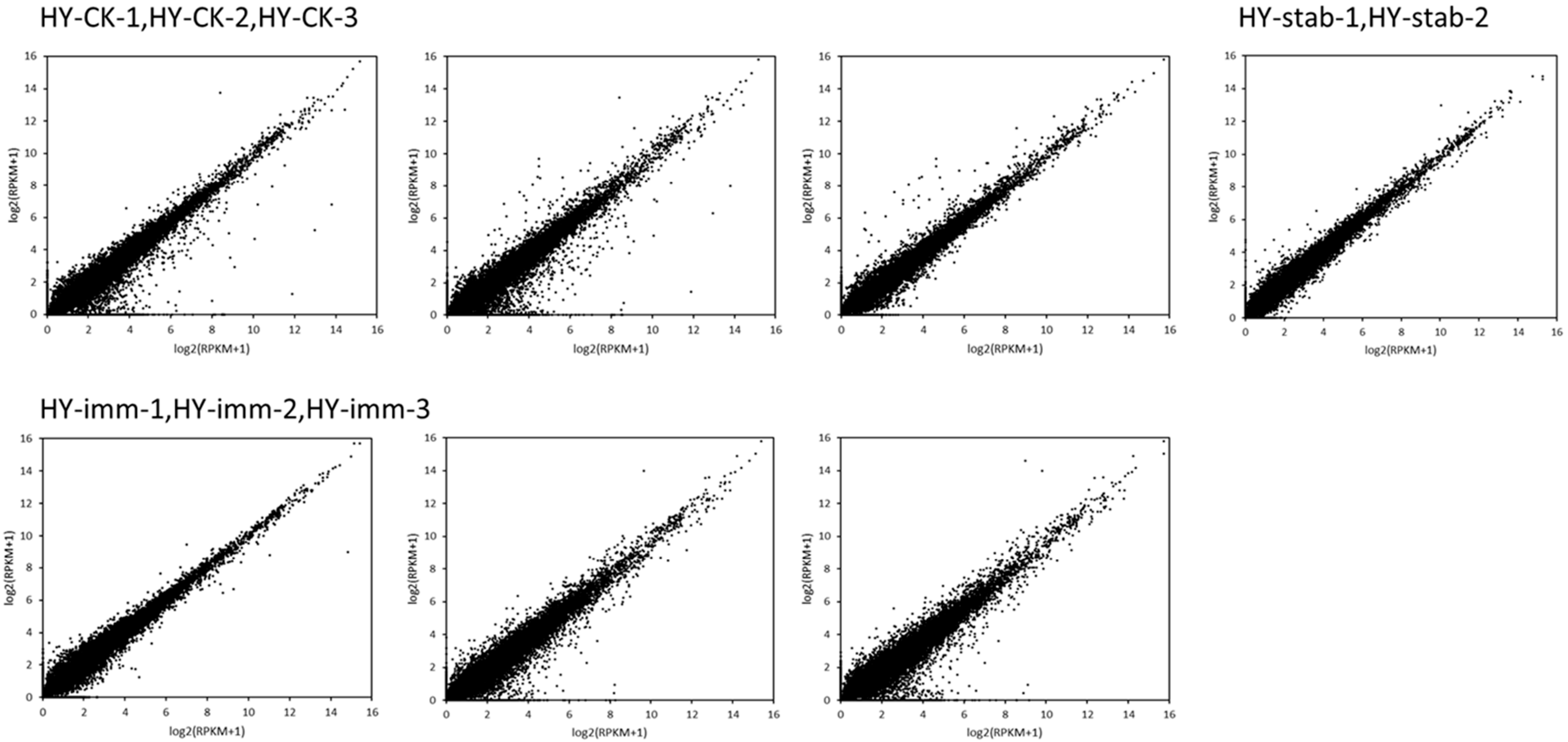

Figure S2. Scatter plot of gene expression of biological repeats.
